# Evaluating the Expression of Oct4 as a Prognostic Tumor Marker in Bladder Cancer

**Published:** 2012

**Authors:** Nasim Hatefi, Nazila Nouraee, Mahmoud Parvin, Seyed-Amir Mohsen Ziaee, Seyed Javad Mowla

**Affiliations:** 1 *Department of Molecular Genetics, Faculty of Biological Sciences, Tarbiat Modares University, Tehran, Iran*; 2 *Department of Pathology, Labbafi-Nejad Medical Centre, Shahid Beheshti University of Medical Sciences, Tehran, Iran*; 3 *Urology and Nephrology Research Centre, Labbafi-Nejad Medical Centre, Shahid Beheshti University of Medical Sciences, Tehran, Iran*

**Keywords:** Bladder cancer, Cancer Stem cell, Oct4, Prognosis

## Abstract

**Objective(s):**

The key transcriptional regulator Oct4 is one of the self-renewal and differentiation-related factors in cancer stem cells, where it maintains "stemness" state. Cancer stem cells have been identified in a variety of solid malignancies. They are a small population of tumor cells with stem cell characteristics, which are a likely cause of relapse in cancer patients. Due to high incidence, mortality, and recurrence rates of bladder cancer and the necessity of accurate prediction of malignant behavior of the tumors, we evaluated the prognostic value of Oct4 expression in formalin-fixed paraffin-embedded (FFPE) tissues of bladder cancer.

**Materials and Methods:**

In this study, Oct4 expression was evaluated in 52 (FFPE) tissues of bladder cancer. RNA extraction from samples of 30 patients from the archive of Labbafi-Nejad Medical Centre in Tehran was performed and Oct4 expression levels were examined by semi-quantitative RT-PCR. The intracellular distribution of Oct4 protein was also determined by immunohistochemistry (IHC).

**Results:**

The results revealed a significant correlation between the expression level of Oct4 and the tumors’ grade and stage. A mostly cytoplasmic distribution of Oct4 protein was also confirmed by IHC.

**Conclusion:**

All together, our data indicate that the expression level of Oct4 gene is correlated with the clinical and histopathological prognostic indexes of tumors and thus can be considered as a potential prognostic tumor marker.

## Introduction

Tumor recurrence and multifocality are two common features of bladder tumors. Moreover, several previous reports suggest that these tumors are derived from a primary transformed progenitor cell (1, 2). Based on Cancer Stem Cell model, CSCs are characterized by self-renewal, heterogeneity (potential for multidirectional differentiation), resistance to apoptosis, and resistance to conventional therapies. Therefore, CSC concept has fundamentally changed our understanding of tumor development and progression as well as the diagnostic and therapeutic approaches. Nowadays, CSCs have been isolated from a variety of solid tumors such as breast cancer, lung, prostate, colon, and brain tumors (3-9). The most important members of CSCs' regulatory core are transcription factors such as Oct4, Sox2, and Nanog, which are defined as key players in the regulatory network for maintaining the “stemness” state of stem cells (8, 10-12). 

Oct4 (POU5f1), a member of POU family, is a transcription factor that is required for pluripotency during early embryogenesis and the maintenance of embryonic stem (ES) cell and pluripotent cell identity. Oct4 expression is strongly repressed following stem cell differentiation (12-17). In somatic cells, Oct4 expresses only in rare sub-populations of multipotent cells with high self-renewal capacity, such as the tissue-specific adult stem cells in normal tissues or cancer stem cells in tumor samples (12, 18, 19). Oct4 has a variety of functions: it can either act as suppressor for genes involved in differentiation or act as a trans-activator for self-renewal genes (20). Based on our previous work, misexpression of Oct4 is  correlated with tumorigenesis and can affect the behavior of tumors such as recurrence or resistance to therapy (10, 15). 

Considering the necessity of using new molecular markers to accurately predict the malignance behavior of bladder tumors, we evaluated the prognostic value of Oct4 expression, as a well-known stem cell molecular marker, in FFPE samples of bladder tumor tissues by means of semi-quantitative RT-PCR and immunohistochemistry (IHC).

## Materials and Methods

***FFPE blocks collection***

FFPE samples (52 blocks of 30 patients) of bladder tumors belonging to patients who had been referred to the Shahid Labbafi-Nejad Medical Centre in Tehran before 2006, were obtained from archival collection of pathology department. After surveying the patients' medical history, samples were selected and categorized according to the histological characteristics based on H&E slides by an expert pathologist. Samples with no or less than 20% of normal tissue presence were selected for the experiments. Moreover, tissues with hemorrhagia were excluded from our analysis due to their low RNA quality (Table 1).

***RNA extraction***

7-10× 10 µm-thick sections of each block were deparaffinized with xylene following rehydration with ethanol. Then tissues were digested with optimized concentration of proteinase K (Fermentase, Vilninus, Lithuania) in order to remove the protein cross links with cellular RNA. Then samples were treated with RNX Plus Solution (Cinnagen, Iran) and RNA extraction procedure were performed according to the manufacturer’s instructions. 

***Reveres transcription-PCR (RT-PCR)***

Due to the existence of processed pseudogenes of Oct4, all extracted total RNAs were treated with RNase-free DNase (Fermentase, Lithuania). For cDNA synthesis, random hexamer primers and RevertAid^TM^ M-MuLV reverse transcriptase (Fermentase, Lithuania) were used according to the manufacturer’s instructions. We also prepared a No-RT control sample for each reaction to detect any potential contamination with genomic DNA.

**Table 1:** A brief clinico-pathological description of patients

No.	Age/Sex	Code and number of block	Grade	Stage	Oct4 expression rate	Radical cystectomy (Date & number of block)	Survival status
1	66/F	79-2453	II	T1	High	No	8/28/2006
2	75/F	84-347	I	T1	Low	No	Alive
3	63/F	79-1258	II	T1	High	No	Alive
4	46/M	79-2698	II	T1	Low	Oct 28, 2012, (2755)	2001
_	79-2755	II	T2a	High			
5	46/M	80-51	I	T1	Low	No	Alive
6	66/M	79-2704	II	T1	High	No	Alive
7	44/M	79-877 I	I T1	T1 0	Low	Dec 10, 2002	Alive
8	53/M	80-1292	I	T1	High	No	1/2/2006
9	81/M	83-1128 I	I Ta	T1 0	Low	No	Alive
10	76/M	81-2693	I	T1	Low	No	Alive
11	52/M	80-121	I	T1	High	No	Alive
12	81/M	81-3619	I	Ta or Tis	High	No	Alive
13	63/F	79-3304	I	T1	Low	No	Alive
		78-2401 III III	II T1 T3b,N1	Ta High High	High	May 21, 2001, (533)	Alive
14 _76/M	80-426						
_	80-533						
15	51/F	78-501 II	II T1	T1 High	High	Partial- Jul 13, 1999-(699)	Alive
		79-909 III III	II T1 T3b	T1 High High	Low	Cystoprostatectomy- Feb 9, 2002-(3263)	2003
16 _66/M	80-2728						
_	80-3263						
17	79/F	83-758	I	Ta	Low	No	Alive
		82-2135 I II	II Ta T1	T1 0 High	0	No	Alive
18 _46/M	83-1109						
_	85-584						
19	68/F	79-1230	I	T1	High	No	Alive
20	58/M	80-1766	I	T1	Low	No	12/14/2003
		84-99 I I I I	I Ta T1 T1 T1	T1 Low Low Low Low	Low	Partial-Apr 26, 2007	Alive
_	84-1386						
21 _66/M	84-2231						
_	85-1257						
_	85-2930						
22	63/M	83-1689 I	II T1	T1 Low	High	No	Alive
23	31/F	85-2238 I	I T1	T1 Low	Low	No	Alive
24	56/M	84-3588 III	II T3b	T1 High	High	Dec 17, 2006, (2401)	Alive
25	74/M	84-1199	II	T1	High	No	Alive
26	67/M	82-2973 I	III Ta	T1 Low	High	No	Alive
27	63/F	85-2027 I	I Ta	Ta Low	0	No	Alive
28	66/F	84-2689 I	I Ta	Ta Low	Low	No	Alive
29	64/M	83-38 I	II Ta	Ta Low	High	No	Alive
30	77/M	79-1407	II	T1	High	No	11/29/2004

F: Female; M: Male

Because of fragmentation of RNA molecules in FFPE samples, we designed specific primers for short segments (<300 bp) of RNA. The specific primers for Oct4 and beta2-microglobin (*ß2m*), as an internal control (accession numbers: NM_002701 and NM_004048, respectively) were designed by Genrunner software (Version 3.05, Hastings Software Inc.) and synthesized by MWG-biotech (Germany), as high-purified salt-free grade. The sequences of the designed primers are as follows: 

beta2-microglobin: 

Forward primer: **5'**- CTA CTC TCT CTT TCT GGC CTG -**3'**

Reverse primer: **5'**- GAC AAG TCT GAA TGC TCC AC – **3'**

These primers amplified a 191 bp segment of human *ß2m* complementary DNA.

Oct4: 

External forward primer: **5'**- TCC CAG GAC ATC AAA GCT CT -**3'**

External reverse primer: **5'**- TCA TTG TTG TCA GCT TCC TCC -**3'**

These primers amplified a 238 bp segment of human Oct4 complementary DNA.

Oct4 nested primers:

Internal forward primer: **5'**- CAT CAA AGC TCT GCA GAA AG -**3'**

Internal reverse primer: **5'**- CTT CCT CCA CCC ACT TCT G -**3'**

The product of amplification of these nested primers is a 217 bp segment.

All designed primers were blasted with human genome to make sure they are not complementary to other regions of the genome (21). In case of β2M, serial dilutions of primary PCR products were used to optimize the amount of template required for the second round without reaching to the threshold level. 

PCR was performed using 2 μl of synthesized cDNA with 0.2 μl of Taq polymerase , as described elsewhere (22). The PCR reaction conditions which were repeated for 37 cycles (*ß2m* and Oct4-round 1) or 30 cycles (Oct4-round 2), were as follows: Initial denaturation at 94°C for 4 min, denaturation at 94°C for 40 sec, annealing at 57°C (*ß2m* and Oct4-round2) or 55 °C (Oct4-round1) for 45 sec, extension at 72°C for 60 sec, and a final extension at 72°C for 10 min.

PCR products were separated by electrophoresis on 1.5% agarose gels, stained with ethidium bromide, and visualized by Gel Documentation (Uvitech, England).

***Statistical analysis***

All experiments were replicated two or three times and the RT-PCR results were analyzed by performing Mann-Whitney and ANOVA tests to determine the difference of Oct4 expression among different groups (SPSS software for windows, version 11, Chicago). Statistical significance was set as* P*< 0.05 and all reported *P*-values were 2-sided.

***Immunohistochemistry***

The IHC procedure was optimized using tissue samples which had been proven to be Oct4-positive by means of RT-PCR in the previous stage. Five µm-thick sections of each block were deparaffinized and rehydrated. For antigen retrieval, tissue sections were boiled in citrate buffer (10 mM, pH 6.0) for 10 min. Endogenous peroxidase activity was blocked with 1.5% H_2_O_2_ for 30 min, and background staining was eliminated with blocking proteins (1% BSA/PBS) for 2 hr. Slides were incubated with anti- Oct4 polyclonal primary antibody (SC-8629; Santa Cruz Biotechnology, USA) diluted 1:100 with 0.1% BSA/PBS solution, overnight at 4°C. Afterwards, tissues were incubated with secondary antibody, anti-goat HRP conjugated (Abcam, USA), overnight at 4°C. Then enzyme development was performed with DAB/H_2_O_2_ complex for 10 min in room temperature and in the absence of light which provides a brownish precipitation. All the stages were similar in negative controls except for the omission of the primary antibody. 

## Results

***Detecting*** ***Oct4*** ***expression in FFPE samples of bladder tumors***

**Figure 1:**
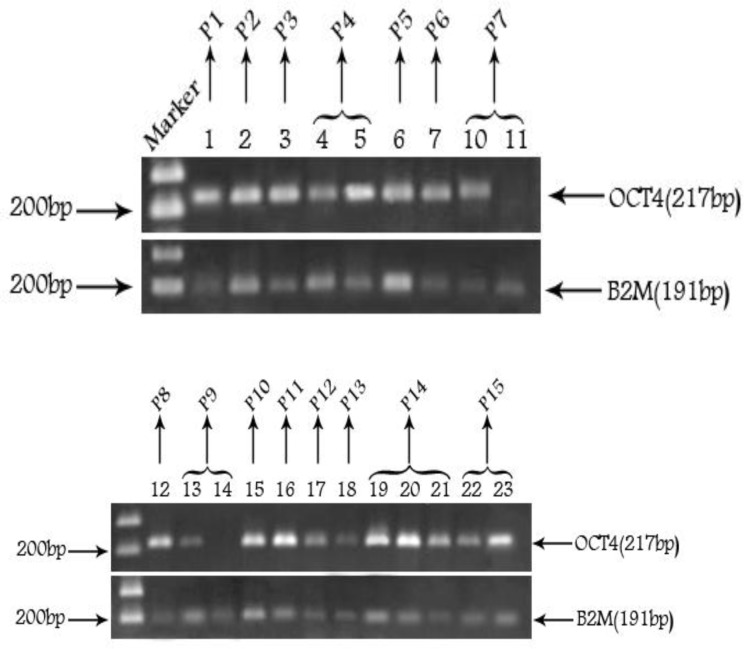
Reverse transcription polymerase chain reaction analysis of the expression of Oct4 and B2M in FFPE samples of 4 patients; consecutive numbers show recurrent samples of the same patient. The 100 bp DNA ladder is used as molecular size marker

We used a semi-quantitative RT-PCR approach to detect Oct4 expression in FFPE samples of bladder and also to compare the level of Oct4 expression among different grades and stages of the tumor samples (Figure 1). Briefly, the intensity of the Oct4 and *ß2m* bands was measured by Uvitech software, and the ratio of Oct4/*ß2m* expression was considered as the intensity of the gene expression. Initially, the median of the expression among all samples were determined and the expression above the median was considered as high and the ones below the median considered as low expression. Among 52 FFPE samples, 23 samples (44%) had high expression, 24 (46%) had low expression, and 5 (10%) had no expression. The samples with no Oct4 expression were classified in a separate group termed as “No expression” group.

**Figure 2:**
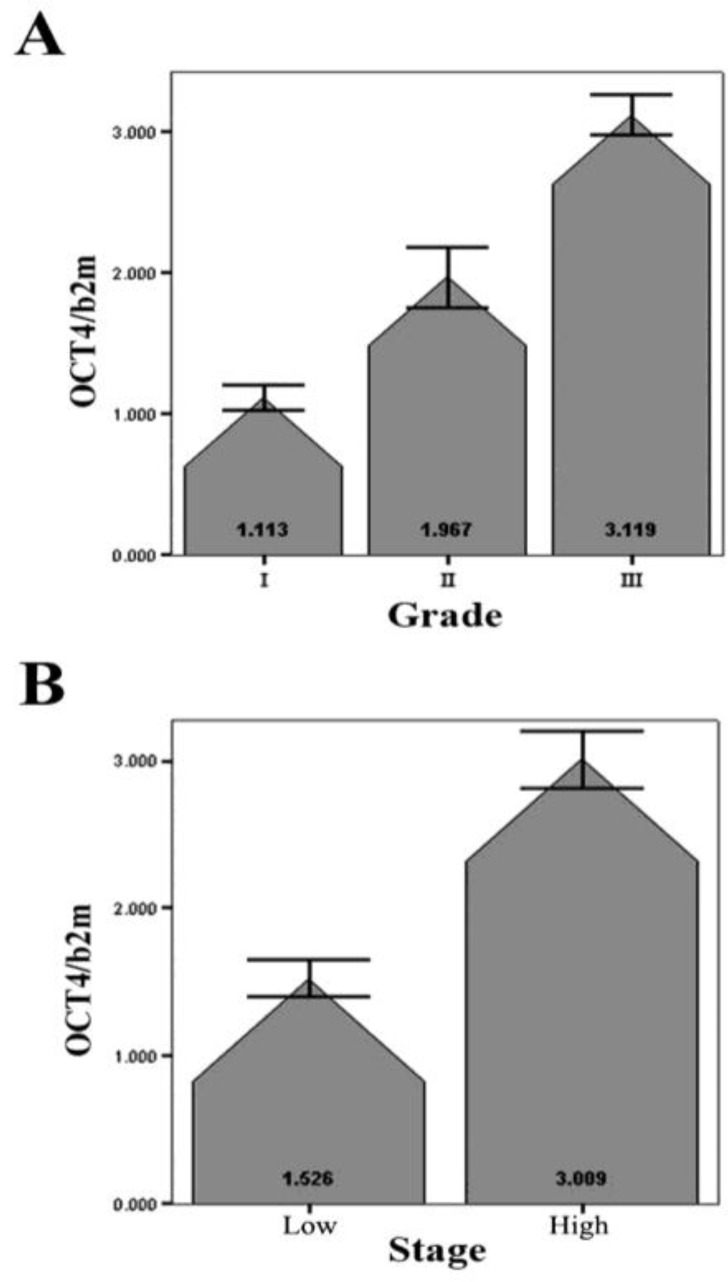
Relative expression of Oct4 in bladder tumors with different grades (A) and stages (B). Values are shown as the mean ±SD. Low stage=stages Tis, Ta, and T1. High stage=stages T2, T3, and T4

One-Way ANOVA test revealed a significant correlation between the average of Oct4 expression and the grade of tumors (*P*< 0.05; Figure 2A). Due to the few number of cases in some stages, we had to classify the samples with Tis, Ta, and T1 stages in one group named “Low stage” and the other samples with T2, T3, and T4 stages in another group named “High stage”. Using the Mann-Witney analysis our data demonstrated a significant correlation (*P*< 0.05) between the expression level of Oct4 and the stage of the tumors (Figure 2B).

***Oct4*** ***is mostly localized within the cytoplasm of tumor cells***

**Figure 3:**
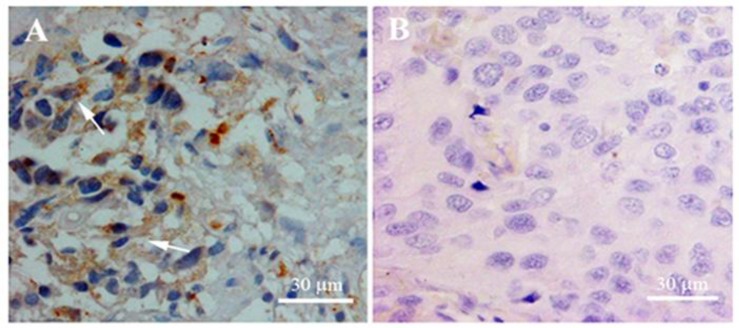
Immunohistochemistry results showing the tissue distribution and subcellular localization of Oct4. Brown signals show the mostly cytoplasmic localization of Oct4 protein (A); Negative control with no primary antibody treatment (B). Slides were counterstained with Hematoxylene and Eosine

Next, we employed IHC to examine whether Oct4 is also expressed at the protein level and also to determine its tissue and subcellular distribution. As it is evident in Figure 3A, there are some Oct4-positive cells in tissue sections showing a cytoplasmic signal for Oct4. However, there is also a rare subpopulation of cells with strong immunoreactivity within their nuclei. There was no immunoreactivity signal within the cells in which the Oct4 antibody was eliminated during IHC (The negative control, Figure 3B), confirming the authenticity of the observed signal for Oct4.

## Discussion

Bladder cancer has high incidence and recurrence rates in Iran; particularly it is the 3^rd^ most frequent cancer in men (23). Thus, early detection and finding reliable methods for screening high risk cases is of vital importance. The current methods of bladder cancer diagnosis are urine cytology and cystoscopy. Urine cytology is a procedure with 95% specificity but low sensitivity, especially in low-grade tumors. Cystoscopy is the current gold-standard method for bladder cancer detection, but it is an invasive and expensive procedure with low specificity and sensitivity in detecting superficial tumors (24). Therefore, there have been lots of efforts in the field to find non-invasive, sensitive, and specific molecular markers for bladder cancer. The molecular markers that could be easily traced in the urine of patients are of special interest (25, 26). The main aim of finding such tumor markers is to provide primarily a way to detect and classify tumors more accurately and ultimately to provide a molecular target for gene-based therapy.

Prompted with the cancer stem cell hypothesis, we have previously reported the expression of a well-known stem cell marker, Oct4, in bladder cancer. Furthermore, the study also revealed a significantly strong correlation between the expression level of Oct4 and the tumor/non-tumor state of the samples (10).

As a stem cell specific transcription factor, Oct4 plays a vital role in pluripotency, self-renewality and prevention of differentiation of inner cell mass (ICM). Based on ours and other reports, Oct4 is also expressed in several tumors including testis, bladder, uterus, breast, and ovarian cancers (10, 27-32). Following unexpected detection of Oct4 in several cancers, it is currently considered as a molecular target for CSC-directed gene therapy. 

In the current study, we have extended our previous study and evaluated the level of Oct4 expression in FFPE archival collections through a retrospective study. Due to the unlimited supply of FFPE samples in most hospitals, the current approach would provide some advantages compared with the works using fresh biopsies or surgical materials. However, in practice, we encountered some problems in following-up the current status of most patients, mostly due to the lack of a good recording system to contact the patients or their families. Therefore, instead of randomly selecting some samples and correlating the Oct4 expression with the clinical outcome of each stage and grade, we had to restrict our work to those patients whom their records and outcomes were available. 

Based on our data, a significant correlation between the expression level of Oct4 and the grade and stage of the samples are evident. These findings are consistent with our previous report suggesting the suitability of Oct4 expression as a molecular marker for the diagnosis of bladder tumors (10). The current investigation also provides some data supporting the suitability of Oct4 as a prognostic molecular marker to predict the malignant nature of bladder cancers. This claim, however, needs to be further examined with a bigger population size comprising a good sample size for each grade and stage subgroups.

In IHC experiment, we detected Oct4 mostly in the cytoplasm of the tumor cells, a finding which is consistent with our previous report on differential expression of Oct4 variants in pluripotent vs. non-pluripotent cell lines (17). The expression of the main variant of Oct4, Oct4A, is restricted in pluripotent cells, where the encoded protein is depicted in the nuclei of the cells. The Oct4B variant differs from the Oct4A by lacking exon 1 and having a bigger exon 2 and is preferentially detected in the cytoplasm of tumor cells. The later finding confirms the identity of the expressed Oct4 in the bladder tumors as Oct4B variant. However, there was a rare subpopulation of cells with nuclear staining for Oct4. The latter cells are probably the cancer stem cells or normal adult bladder stem cells which reside within the tumor tissues. 

All together, our data provide strong evidence to support the correlation of Oct4 expression with the malignant behavior of bladder cancer.

## Conclusions

When these data are taken together, our study for the first time demonstrates a differential expression pattern of Oct4 in FFPE tissues of bladder cancer with different progression states. This could potentially have a practical usefulness in prognosis and/or therapy of the tumor.
